# Supplementary material for: Functional Characterization of 21 Rare Allelic CYP1A2 Variants Identified in a Population of 4773 Japanese Individuals by Assessing Phenacetin O-Deethylation
Source: J Pers Med. 2021 Jul 22;11(8):690. doi: 10.3390/jpm11080690 (PMC8401128; doi:10.3390/jpm11080690)
Supplement: Supplementary file 1 [file jpm-11-00690-s001.zip › jpm-1276540-supplementary.pdf]

Supplementary Materials

Functional Characterization of 21 Rare Allelic CYP1A2 Variants Identified in a Population of 4773 Japanese Individuals by Assessing Phenacetin O-deethylation

Masaki Kumondai, Evelyn Marie Gutiérrez Rico, Eiji Hishinuma, Yuya Nakanishi, Shuki Yamazaki, Akiko Ueda, Sakae Saito, Shu Tadaka, Kengo Kinoshita, Daisuke Saigusa, Tomoki Nakayoshi, Akifumi Oda, Noriyasu Hirasawa, and Masahiro Hiratsuka

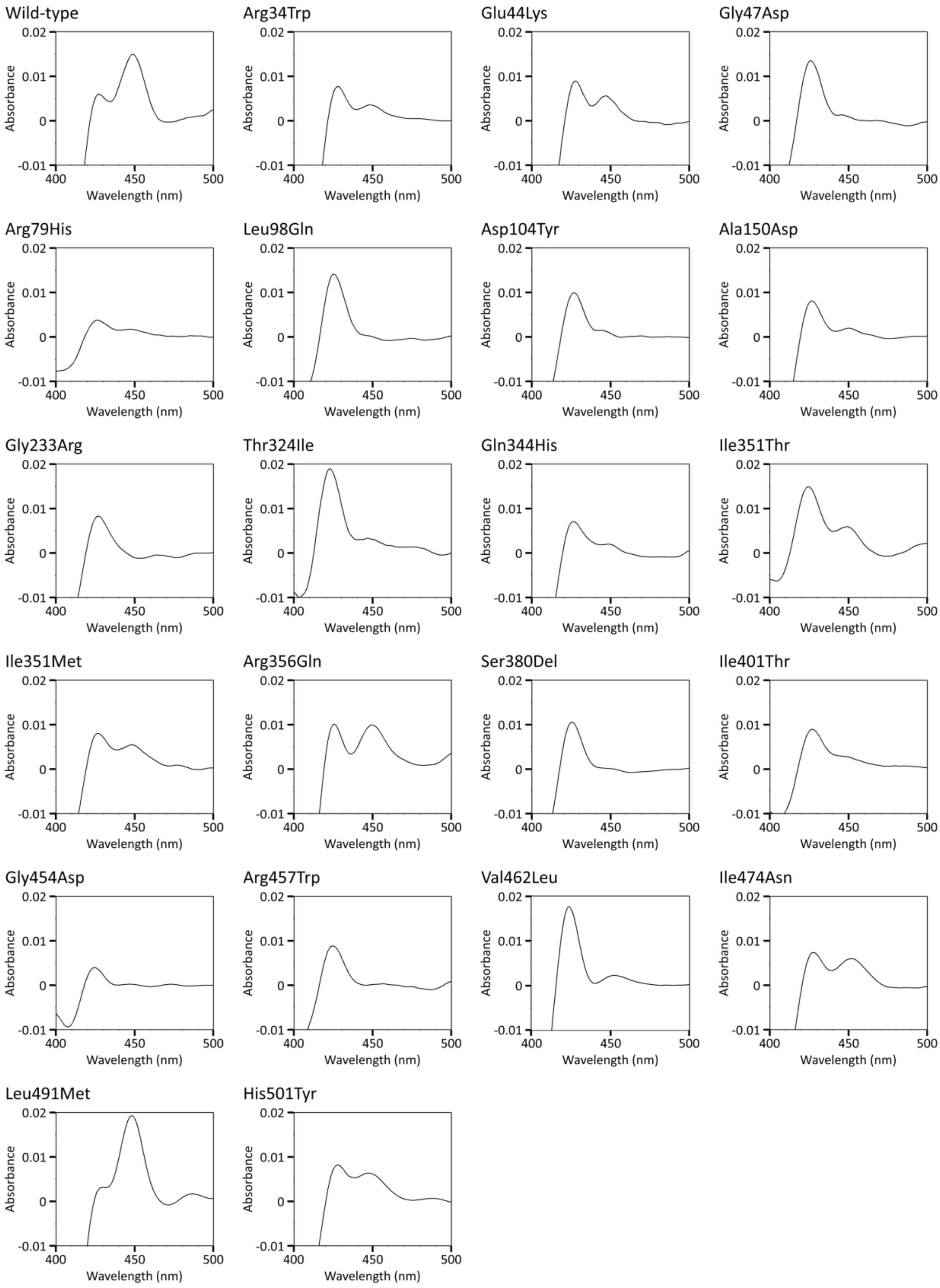

Supplementary Figure S1. Representative CO-difference spectra of CYP1A2 variant proteins expressed in 293FT cells. All assays and measurements were performed in triplicate using a single microsomal preparation. Mock 1 represents transfection with 10 μg mock plasmid. Mock 2 represents transfection with 9.6 μg mock plasmid, 0.2 μg CPR plasmid, and 0.2 μg cytochrome b_5 plasmid.

Supplementary Table S1. PCR primers used to amplify sequences of the human *CYP1A2* gene.

Exon	Size (bp)	Forward primer (5′-3′)	Reverse primer (5′-3′)
2	1203	GGAATCTTGAGGCTCCTTTCC	TCCAGGTCACACAGCTGGTC
3	432	AGACCAAGTTGGGAGGATAGG	AGGACTTTGCTGTTTCTCCAC
4-5	707	GGCCAGAGAAAGCTAATGCTG	GATCTGTGGGGAACGAGGTC
6	344	TTCTACCTCTTCCCTGTTCC	TAGCAATTGAGGTCCCCTCC
7	439	GGTCCCATCTCCTCTGTTCC	CCTGCACTTGGCTAAAGCTG
